# Differential inhibition of adenylylated and deadenylylated forms of *M*. *tuberculosis* glutamine synthetase as a drug discovery platform

**DOI:** 10.1371/journal.pone.0185068

**Published:** 2017-10-03

**Authors:** A. Theron, R. L. Roth, H. Hoppe, C. Parkinson, C. W. van der Westhuyzen, S. Stoychev, I. Wiid, R. D. Pietersen, B. Baker, C. P. Kenyon

**Affiliations:** 1 CSIR Biosciences, Pretoria, South Africa; 2 School of Biomedical Sciences, Charles Sturt University, Orange NSW, Australia; 3 DST-NRF Centre of Excellence for Biomedical Tuberculosis Research, SAMRC Centre for Tuberculosis Research, Division of Molecular Biology and Human Genetics, Faculty of Medicine and Health Sciences, Stellenbosch University, Tygerberg, Cape Town, South Africa; University of Padova, Medical School, ITALY

## Abstract

Glutamine synthetase is a ubiquitous central enzyme in nitrogen metabolism that is controlled by up to four regulatory mechanisms, including adenylylation of some or all of the twelve subunits by adenylyl transferase. It is considered a potential therapeutic target for the treatment of tuberculosis, being essential for the growth of *Mycobacterium tuberculosis*, and is found extracellularly only in the pathogenic *Mycobacterium* strains. Human glutamine synthetase is not regulated by the adenylylation mechanism, so the adenylylated form of bacterial glutamine synthetase is of particular interest. Previously published reports show that, when *M*. *tuberculosis* glutamine synthetase is expressed in *Escherichia coli*, the *E*. *coli* adenylyl transferase does not optimally adenylylate the *M*. *tuberculosis* glutamine synthetase. Here, we demonstrate the production of soluble adenylylated *M*. *tuberulosis* glutamine synthetase in *E*. *coli* by the co-expression of *M*. *tuberculosis* glutamine synthetase and *M*. *tuberculosis* adenylyl transferase. The differential inhibition of adenylylated *M*. *tuberulosis* glutamine synthetase and deadenylylated *M*. *tuberulosis* glutamine synthetase by ATP based scaffold inhibitors are reported. Compounds selected on the basis of their enzyme inhibition were also shown to inhibit *M*. *tuberculosis* in the BACTEC 460TB™ assay as well as the intracellular inhibition of *M*. *tuberculosis* in a mouse bone-marrow derived macrophage assay.

## Introduction

Tuberculosis (TB) is a worldwide pandemic, caused by infection with the bacterium *Mycobacterium tuberculosis*. Although current treatment can be effective, existing drugs must be taken for at least six months to prevent relapse. Poor treatment compliance contributes directly to the emergence of multidrug- and extensively drug-resistant (MDR and XDR) strains of *M*. *tuberculosis*. New targets for drugs are therefore required. These new drugs should also simplify and shorten the treatment period, as well as reduce drug-drug interactions in patients co-infected with HIV. Drug discovery programmes for infectious diseases are normally focussed on pathogen proteins whose function is known to be essential to the bacterial cell, combined with a lack of mammalian homologues. One such potential drug target for TB is adenylylated glutamine synthetase (GS).

GS (EC 6.3.1.2) catalyzes the reversible conversion of l-glutamic acid, ATP and ammonia to l-glutamine, ADP and inorganic phosphate via a γ-glutamyl phosphate intermediate [1]. It is a central enzyme in nitrogen metabolism, and can be regulated by at least four different mechanisms: (a) adenylylation and deadenylylation of a conserved tyrosine residue, (b) conversion between a relaxed (inactive) and taut (active) state depending on the divalent metal cation present, (c) cumulative feedback inhibition by multiple end products of glutamine metabolism, and (d) repression and derepression of GS biosynthesis in response to nitrogen availability [1].

Three distinct forms of GS occur, with GS-I found only in bacteria (eubacteria) and archaea (archaebacteria) [2]. GS-II occurs only in eukaryotes, and soil-dwelling bacteria, while GS-III genes have been found only in a few bacterial species. Two significant prokaryotic GS-I sub-divisions exist: GS-Iα and GS-Iβ [3]. The GS-Iβ enzyme is regulated via the adenylylation/deadenylylation cascade, which does not occur in the GS-Iα or GS-II sub-divisions. *M*. *tuberculosis* and *Escherichia coli* GS are regulated in this manner, while the human homologue belongs to GS-II and is not subject to adenylylation, a difference that can be exploited by developing drugs that are only active against the adenylylated form of the enzyme.

The extent of adenylylation of the *E*. *coli* GS is regulated in response to the intracellular concentrations of 2-ketoglutarate and glutamine, via the reversible adenylylation of a tyrosine residue (Tyr397) in each subunit of GS [1, 4–8]. The presence of adenylylated GS predominates in a nitrogen-rich, carbon-limited media, while the deadenylylated form tends to predominate under conditions of nitrogen limitation [1, 4–15]. The regulation of the adenylylation state of GS is accomplished by three proteins: (1) uridylyltransferase/uridylyl-removing enzyme, (2) the signal transduction protein P_II_, and (3) adenylyltransferase or ATase. High intracellular concentrations of glutamine activate the uridylyl-removing enzyme, which causes the deuridylylation of P_II_. This interacts with the ATase, which then catalyses the adenylylation of the GS. A high intracellular 2-ketoglutarate concentration activates uridylyltransferase, which transfers UMP to each subunit of P_II_, forming P_II_-UMP. The P_II_-UMP interacts with the Atase, which in turn catalyses the removal of AMP from the GS. Research on the effect of glucose, ammonia and glutamic acid concentrations has shown that the adenylylation state of GS is a function of metabolic flux rather than absolute concentration only [10]. The activity of GS is therefore regulated by both the nature and the availability of the ammonia source [1,8]. The current view is that the level of GS activity is inversely related to the degree of adenylylation [reviewed in 1, 9, 10] and that adenylylated residues may be present on any number of subunits from zero to 12, depending on carbon and nitrogen availability [13, 16–21]. GS is therefore responsible for the assimilation of ammonia when the available ammonia in the environment is restricted, as well as for the formation of glutamine for the synthesis of protein and other nitrogen compounds. In ammonia-rich medium, the level of GS is low and GS functions primarily for the synthesis of glutamine.

A number of factors make GS a potential drug target in the fight against TB, including being considered essential for the survival of *M*. *tuberculosis* [22–25]. The GS inhibitor l-methionine-*S*,*R*-sulphoximine (MSO) inhibits growth of *M*. *tuberculosis* both *in vitro* and *in vivo* [22,23]. It is located extracellularly, a characteristic that is found only in the pathogenic mycobacteria such as *M*. *tuberculosis* and *Mycobacterium bovis*, and not with the non-pathogenic strains of *Mycobacterium smegmatis* and *Mycobacterium phlei* [21,22]. This location means a potential drug does not have to pass the *M*. *tuberculosis* cell wall barrier. It appears to play an important role in cell wall biosynthesis, in the form of a cell wall component found only in pathogenic mycobacteria: poly-l-glutamate/glutamine [26, 27].

*M*. *tuberculosis* GS (*Mtb*GS*)* has previously been successfully expressed in heterologous systems including the non-pathogenic mycobacterial strain *M*. *smegmatis* and *E*. *coli* [28–30]. Mehta *et al* expressed *M*. *tuberculosis* GS in *E*. *coli* host strains that were deficient in either chromosomal GS, or both chromosomal GS and ATase [30]. They found that the *E*. *coli* ATase was inefficient in adenylylating the heterologous *M*. *tuberculosis* GS, with only ~25% of subunits being modified. A lack of *E*. *coli* ATase yielded completely deadenylylated *M*. *tuberculosis* GS. As a result no crystal structure exists for *M*. *tuberculosis* fully adenylylated GS [31]. A number of studies have been undertaken targeting *Mtb*GS as a potential therapeutic target however, in none of these investigations was an attempt made to exploit the dichotomy between adenylylated and deadenylylated GS [32–39]. As outlined prokaryotic GS is regulated via a complex cascade that is based on the availability of NH_4_^+^ and glucose to the organism and the intracellular concentrations of 2-ketoglutarate and glutamine [1, 4–8]. This regulation results in the adenylylation or deadenylylation of the GS with a concomitant switch in the enzymes affinity from Mn^2+^ to Mg^2+^[1, 4–8, 30]. The adenylylation and the switch in metal ion specificity significantly impacts on the enzyme activity of GS and therefore probably impacts on the structure of the active site. It is proposed that this dichotomy may be exploitable in creating GS inhibitors that target only prokaryotic GS as only bacterial GS have regulation via the adenylylation/deadenylylation cascade.

Here, we describe the production of both the deadenylylated and adenylylated forms of *Mtb*GS in *E*. *coli*. Deadenylylated *Mtb*GS is produced by constitutive expression of *Mtb*GS in an *E*. *coli* strain deficient in both *E*. *coli* GS and ATase activities, while adenylylated *Mtb*GS is produced when co-expressed with an inducible *M*. *tuberculosis* ATase. Adenylylation was measured using the γ-glutamyl transferase assay, mass spectrometry and determination of phosphate content. IC_50_ values of the known GS inhibitors MSO and phosphinotricin (PhosT) were also determined. A battery of ATP scaffold compounds were identified and screened for their differential inhibitory effect on adenylylated *Mtb*GS and deadeylylated *Mtb*GS. Two of these compounds showed micromolar activity against *Mtb*GS, acceptable activity against a cell-free and macrophage model of *M*. *tuberculosis* indicating their possible druggability. The two compounds identified here represent a good starting point for a hit-to-lead campaign to develop selective, druggable agents capable of selectively inhibiting the adenylated form of *Mtb*GS in view of identifying novel agents against *M*. *tuberculosis* infection.

## Experimental procedures

### Plasmids and bacterial strains

*E*. *coli* JM109 (Promega Corporation) was used for cloning. Restriction endonucleases were purchased from Fermentas Life Sciences and Ex Taq^TM^ DNA polymerase from TaKaRa Bio Inc.

*M*. *tuberculosis* glutamine synthetase *glnA* gene was PCR amplified from genomic DNA of *M*. *tuberculosis* H37Rv (ATCC 25618) using the oligonucleotide primers TB1: 5’-GATGGATCCACCCGATAACCAG-3’ and TB2: 5’-GATGGATCCTCGAAAAACCTCG-3’. The amplified DNA was then digested with *Bam*HI (sites underlined in primer sequences) and ligated with similarly digested pBluescript SKII^+^, generating plasmid pTBSK, with the *glnA* gene under contol of the constitutive T3 promoter.

The *M*. *tuberculosis* H37Rv adenylyl transferase *glnE* gene was PCR amplified from genomic DNA using the oligonucleotide primers TBglnE-8: 5’-TAGCATATGGTCGTGACCAAAC-3’ and TBglnE-9: 5’-CAGGATCCTTAACTCCCGAACAC-3’. The amplified DNA was then co-digested with *Nde*I and *Bam*HI (sites underlined in primer sequence) and ligated with *Nde*I and *Bgl*II digested pCDFDuet-1 (Novagen) to construct pTBGlnE, with *glnE* downstream of the IPTG-inducible T7*lac* promoter.

For *Mtb*GS expression, the chromosomal adenylyl transferase *glnE* gene of the glutamine synthetase auxotroph YMC11 was deleted using the Quick and Easy *E*. *coli* Gene Deletion Kit (Gene Bridges, GmbH) to create the strain YMC11E [40]. The Novagen λDE3 Lysogenization Kit was used to create YMC11E(DE3).

### Production of adenylylated and deadenylylated *Mtb*GS

Deadenylylated *Mtb*GS was expressed in YMC11E containing pTBSK. This strain was inoculated in 50 ml M9ZB medium (1% w/v N-Z-Amine A; 85 mM NaCl) containing M9 Salts (22 mM Na_2_HPO_4_; 22 mM KH_2_PO_4_; 18 mM NH_4_Cl; 8.5 mM NaCl) and supplemented with 100 μg.ml^-1^ ampicillin, 1mM MgSO_4_ and 4% (w/v) glucose. The inoculum was grown at 28°C for 16 hours with shaking at 220 rpm. Subsequently, 5 ml of the culture was transferred to 250 ml of the same medium, which was grown at 28°C for 16 hours. The cultures were harvested by centrifugation for 10 min at 16,300*g* (4°C) and the bacterial pellet used for *Mtb*GS purification.

Adenylylated *Mtb*GS was produced using YMC11E(DE3) containing both pTBSK and pTBglnE. This strain was inoculated into 50 ml of the same medium as above, including 50 μg.ml^-1^ streptomycin. The cultures were incubated at 33°C for 16 hours with shaking at 220 rpm. Thereafter, 5 ml was transferred to 250 ml of the same medium and grown at 33°C for a further 8 hours. *M*. *tuberculosis* adenylyl transferase expression was then induced by the addition 1 mM IPTG. After a further incubation at 33°C for 16 hours with shaking at 220 rpm, the cultures were harvested by centrifugation for 10 min at 16,300*g* (4°C) and the bacterial pellet used for *Mtb*GS purification.

### Purification of adenylylated and deadenylylated MtbGS

The purification of adenylylated and deadenylylated *MtbGS* from a cell free extract was carried out in a three stage process; a streptomycin sulphate precipitation to remove contaminating nucleic acid, an anion-exchange chromatography stage which removed the major fraction of contaminating proteins and an AMP-Sepharose affinity chromatography”polishing” stage which produced the purified enzyme (See Supporting Information for detail, S1 File).

### Production and purification of adenylylated and deadenylylated *E*. *coli* GS

For comparison purposes, adenylylated and deadenylylated *E*. *coli* GS was also heterologously expressed and purified. Recombinant deadenylylated *E*. *coli* GS was produced in an *E*. *coli* strain lacking chromosomal *glnA* (GS) and *glnE* (ATase) genes. Adenylylated *E*. *coli* GS was produced in a strain lacking chromosomal *glnA* (GS) and *glnD* (uridilyl transferase) genes (See Supplementary Information for detail, S1 File) [41, 42]. Sodium dodecyl-sulphate polyacrylamide gel electrophoresis (SDS-PAGE) was used to analyse the molecular mass and purity of the isolated enzymes [43]. Protein concentrations were determined by using the Quant-IT^TM^ Protein Assay Kit (Invitrogen, USA) used in conjunction with the QUBIT^TM^ fluorometer.

### Determination of degree of adenylylation of purified GS

#### Mass spectrometry

A 40 μl aliquot of purified GS in 10 mM imidazole pH 7.0 was loaded on an OPTI-LYNX C4 trap cartridge at 100 μl/min using 2% ACN (Acetonitrile)/0.1% FA (Formic Acid). Samples were eluted using linear acetonitrile gradient (2–90% ACN/5% FA) in 5min and TOF-MS spectra, in the range 700–1700 mz, collected using QSTRA-Elite mass spectrometer with TurboIon source installed. The multiply charged series of TBGS was deconvoluted by the Bayesina Protein Recostruct tool of Bioanalyst QS 2.0 using a mass range of 40–70kD and signal to noise threshold of 3.

#### Hydrolysis of GS for determination of phosphate content

The relative phosphate content of the purified GS proteins was determined by hydrolysis to release the phosphate [44]. Purified GS (4 to 8 nmol in 1 ml) was digested by the addition of 4 M HCl (1 ml), and the mixture was evaporated to dryness. The residue was resuspended in 2 M HCl (1 ml) plus 20 μl of H_2_O_2_ (30% v/v), and the mixture was again evaporated to dryness. Five sequential additions of H_2_O_2_ (200 μl) with evaporation to dryness were used to complete the hydrolysis. The residue was resuspended in Milli-Q water (1 ml), and phosphate content was determined by using the *BioVision* Phosphate colorimetric assay kit as per supplier’s manual.

#### γ-Glutamyl transferase assay

The GS γ-glutamyl transferase enzyme activity was used to calculate the degree of adenylylation of the purified GS using the standard method as outlined by Shapiro and Stadtman [41].

#### Dose response assays: MtbGS inhibitor screening and specific activity determination

Standard GS inhibitors methionine sulphoximine (MSO), phosphinotricin (PhosT) and a battery of 213 compounds were tested for inhibition of the adenylylated and dedenylylated forms of *Mtb*GS. Serial 4-fold dilutions of MSO and PhosT were prepared in DMSO. Two dilution series were set up, one starting at 1 mM and the other at 2 mM. Eight dilutions were performed in DMSO, to produce a concentration range of 1 mM– 61 nM or 2 mM—122 nM. Twenty μl of each dilution was distributed into duplicate wells of the reaction plate. Each well received 164 μl *Mtb*GS in 50 mM HEPES, 4 mM NH_4_Cl, 1.8 mM MnCl_2_ or MgCl_2_. For adenylylated *Mtb*GS, HEPES pH 6.95 and MnCl_2_ were used, while for deadenylylated *Mtb*GS, HEPES pH 7.15 and MgCl_2_ were required. The plates were incubated at 37°C for 2 hours, after which the reactions were initiated by adding sodium glutamate and ATP to final concentrations of 4 mM and 0.8 mM respectively, with a final reaction volume of 200 μl. After further 2 hour incubation, the reactions were terminated by the addition of 1 μl 50% trichloro acetic acid to each well. Blank wells (no enzyme) were also prepared for each individual compound. In addition, each plate contained control wells (10% DMSO without inhibitor) and blank control wells (10% DMSO, no inhibitor, no enzyme). After termination of the reactions, ADP levels in each well were determined by HPLC.

#### HPLC based enzyme assay

The enzyme activity of GS was determined by measuring the ADP concentration by HPLC [45]. Samples were analysed on a Hewlett Packard series 1100 HPLC fitted with a Luna 5μm C18 column. Each sample was automatically injected (2 μl) and separated with a mobile phase containing 51 mM KH_2_PO_4_, PIC A Low UV Reagent, 25% (v/v) acetonitrile. An AMP, ADP and ATP standard was used to calibrate the HPLC and the concentration of ADP in each sample was determined by the area under the curve using Agilent Technologies ChemStation software. The ADP values in the blank wells were subtracted from enzyme-containing wells, and percentage enzyme activity in each well calculated relative to the average net ADP values of the control wells without inhibitor. The enzyme activity was expressed as a percentage of the maximum enzyme activity in the absence of any inhibitor.

#### HeLa cell cytotoxicity assay

HeLa cells (Human Negroid cervix epitheloid adenocarcinoma, ECACC) were routinely maintained as monolayer cell cultures in Eagle's minimal essential medium (EMEM) containing 5% fetal bovine serum, 2 mM L-glutamine and 50 μg/ml gentamicin at 37°C in a 5% CO_2_ incubator. To perform the cytotoxicity assay, the cells (3–19 passages) were used to inoculate 96-well microtiter plates at plating densities of 7000 cells/well. After incubating for 24 hours, the culture medium was replaced with medium containing serial dilutions of the experimental compounds. Each dilution series consisted of 8 x 3-fold serial dilutions, spanning the final concentration range 100–0.05 μM. Triplicate wells were used for each concentration point (n = 3). The dilutions were prepared from compound stocks of 10 mM in DMSO, thus the final DMSO content in the highest compound concentration wells was 1%. Control wells consisted of cells incubated in medium with 1% DMSO, while blank wells contained medium without cells. Emetine was used as a positive control drug standard. The plates were incubated for 48 hours after addition of the compounds. The cellular protein present after the incubation period was fixed to the bottom of each well with cold 50% TCA, washed in tap water and stained with 0.4% sulforhodamine B (SRB) in 1% acetic acid. Unbound dye was removed by washing with 1% acetic acid, after which protein-bound dye was solubilised with 10 mM Tris base and transferred to a duplicate 96-well plate. Optical density was measured at 540 nm using a Tecan Infinite F500 multiwell spectrophotometer. OD_540_ values of the blank wells were subtracted from the readings obtained for all the other wells, and percentage cell viability at each test compound concentration calculated relative to the untreated (DMSO alone) control wells. Percentage viability was plotted against Log (compound concentration) and the IC_50_ for each compound calculated from fitted non-linear regression dose-response curves using GraphPad Prism software.

### Testing of compounds against M.tb strains in-vito and ex-vivo using the BACTEC 460TB™ assay

#### Bacterial strains

All *M*.*tb* strains used were from a strain bank kept in the Division Molecular Biology and Human Genetics of of Stellenbosch University. *M*.*tb* H37Rv reference strain (ATCC 25618) and a clinical isolate of *M*.*tb* (Beijing220) were used to evaluate compounds for anti-tuberculosis activity. The H37Rv strain was sensitive to the breakpoint concentrations (approximately 10x higher than their minimal inhibitory concentrations (MIC) of isoniazid (0.25ug/ml), ethambutol (9.4ug/ml), and rifampicin (2.0ug/ml) [46]. However, the Beijing220 clinical isolate was resistant to isoniazid and rifampicin [47, 48].

#### Test compounds

The compounds identified were used in the BACTEC 460TB™ assay [49]. The identified compounds were dissolved and diluted with 100% DMSO.

#### Bacterial selection

All mycobacterial colonies were cultured and selected from Lowenstein-Jensen slant [50] cultures followed by culture in Middlebrook 7H9 mycobacterial growth medium supplemented with OADC (0.005% v/v oleic acid (Merck), 0.5% w/v BSA Fraction V, 0.2% v/v glucose, 0.02% v/v catalase (Merck), w/v 0.085% NaCl). Cultures were stained by acid-fast staining (Ziehl-Neelsen) to control for contamination.

#### BACTEC 460TB™ system determination of mycobacterial growth

The BACTEC 460TB™ system has been devised to monitor mycobacterial growth of the slow growing species. The bacteria are grown on a radioactive substrate and the radioactive carbon dioxide produced is directly proportional to the mycobacterial growth rate. Read-out values are expressed as growth index (GI). *M*.*tb* reference strain H37Rv was cultured in 7H9 mycobacterial medium (Difco) enriched with ADC (0.5% w/v BSA Fraction V, 0.2% v/v glucose, 0.015% v/v catalase (Merck) with continuous stirring at 37°C. When cultures reached a density of approximately 0.16 at OD_600_ (one McFarland), 0.1 ml was inoculated into a BACTEC 12B medium vial. These primary cultures were incubated at 37°C until a growth index of 500 (± 50) was reached. These primary cultures were used for drug testing of known and unknown compounds. Compounds, resuspended in DMSO, were sterilized through a 13 mm organic solvent resistant syringe filter with 0.22 micron pore size (Millex-LG). Undiluted and sequentially diluted samples were tested for growth inhibitory activity. 0.1 ml of primary culture and 0.1 ml drug compound were added to a BACTEC 460TB™ vial, the vials incubated at 37°C, and the growth monitored every 24 hours. Controls included cultures with and without compound solvent. GI readings were continued until the controls reached the maximum GI value of or below 999. Control GI values between 50 and 800 are normally used to evaluate the efficacy of compounds with possible anti-tuberculosis activity.

### Testing of screening hits against *M*.*tb* in a macrophage assay

#### *M*.*tb* cultures

*M*.*tb* clinical MDR strain (Beijing220) was cultured in 7H9 broth supplemented with 10% oleic acid-albumin-dextrose catalase (OADC, Difco, BD Biosciences, Mountain View, CA, USA) and 2% glycerol and 0.05% Tween 80 (Glickman et. al., 2000). Liquid cultures were grown for up to 3 weeks and stored at -80°C in 1ml aliquots with 15% glycerol. Clumps were eliminated by 30 passages through a needle (26-gauge 3/8; 0.45 x 10 for intradermal injection; BD Biosciences, USA). Before infection, viability of mycobacteria was evaluated by the propidium iodide exclusion method to ensure >90% viability. Contamination was checked by the Ziehl-Neelsen stain. The required amount of mycobacteria was spun down at 16 300xg for 5 min. The supernatant was removed and the bacteria resuspended in PBS (phosphate buffered saline) and passaged again 20 times and then used for macrophage infection.

#### Manipulation of mouse bone marrow-derived macrophages (MBMM)

This research study was approved by the Stellenbosch University Animal Ethics committee on Animal Care and Use and complies with the South African Animal Protection Act (Act no 71, 1962). Animal Ethics No. SU-ACUD14-00041. Mice were supplied by the Animal House of the University of Stellenbosch, South Africa. Mice were cared for in accordance with ethical laws on animal manipulation. Untreated mice were killed by cervical dislocation inside the Animal House 10 minutes after being received. Bone marrow cells were isolated from the femurs and tibias and differentiated into macrophages as previously described [51]. Bone marrow cells were obtained from femurs of 6–8 week-old C57BL/6 female mice and seeded into 24-well tissue culture plates. The culture medium was RPMI-1640 (Sigma, USA) supplemented with 10% heat-inactivated fetal bovine serum (Gibco), 10% L-929 cell conditioned medium (a source of colony stimulating factor-1) with no antibiotics. Incubation was performed at 37°C, 5% CO_2_. At 5 days after seeding, adherent cells were washed twice with RPMI and re-fed with complete medium. Medium was then renewed every second day.

#### Macrophage infection and harvesting of TB

Frozen aliquots of *M*.*tb* (Beijing220) were removed from the -80°C freezer where they were kept for long-term storage and thawed and processed as indicated. Infection of the 7-Day old MBMM in 24-well plates was performed in triplicates with 100 μl of bacterial suspension relative to a multiplicity of infection (MOI) of 2, and incubated for 4 hrs at 37°C, 5% CO_2_. After the 4 hr incubation period, non-ingested *M*.*tb* (Beijing220) was removed by washing 4 times with ice-cold PBS. Fresh RPMI medium (1 ml) was added and again on day 2. At day 4 post infection the test compounds were added at the desired concentrations between 10 μM and 100 μM. Included were uninfected, infected (no drug) and an isoniazid drug controls. On day 2 post drug intervention the macrophages were washed 3 times with cold PBS (4°C) and then the bacteria harvested by adding 1 ml 0.025% SDS for 5 min to lyse the adherent macrophages. The contents of each well were placed into separate Eppendorf tubes and the bacteria were pelleted at 13000 rpm for 5 min. The bacteria was resuspended in 100 μl 7H9 Middlebrook medium and then inoculated into BACTEC 460TB™ vials. Growth was monitored over time until the BACTEC 460TB™ readings of the infected (no drug) control reached about 500. The percentage growth inhibition for each drug compound was determined by relating the linear growth unit readings to the infected (no drug) control.

## Results

### Production and purification of adenylylated and deadenylylated MtbGS

Expression strain *E*. *coli* YMC11E was constructed by deleting the chromosomal *glnE* gene in the GS auxotroph YMC11. For induction of *M*. *tuberculosis* ATase expression from pTBGlnE, the strain was lysogenised with λDE3 to create YMC11E(DE3).

**Fig 1:**
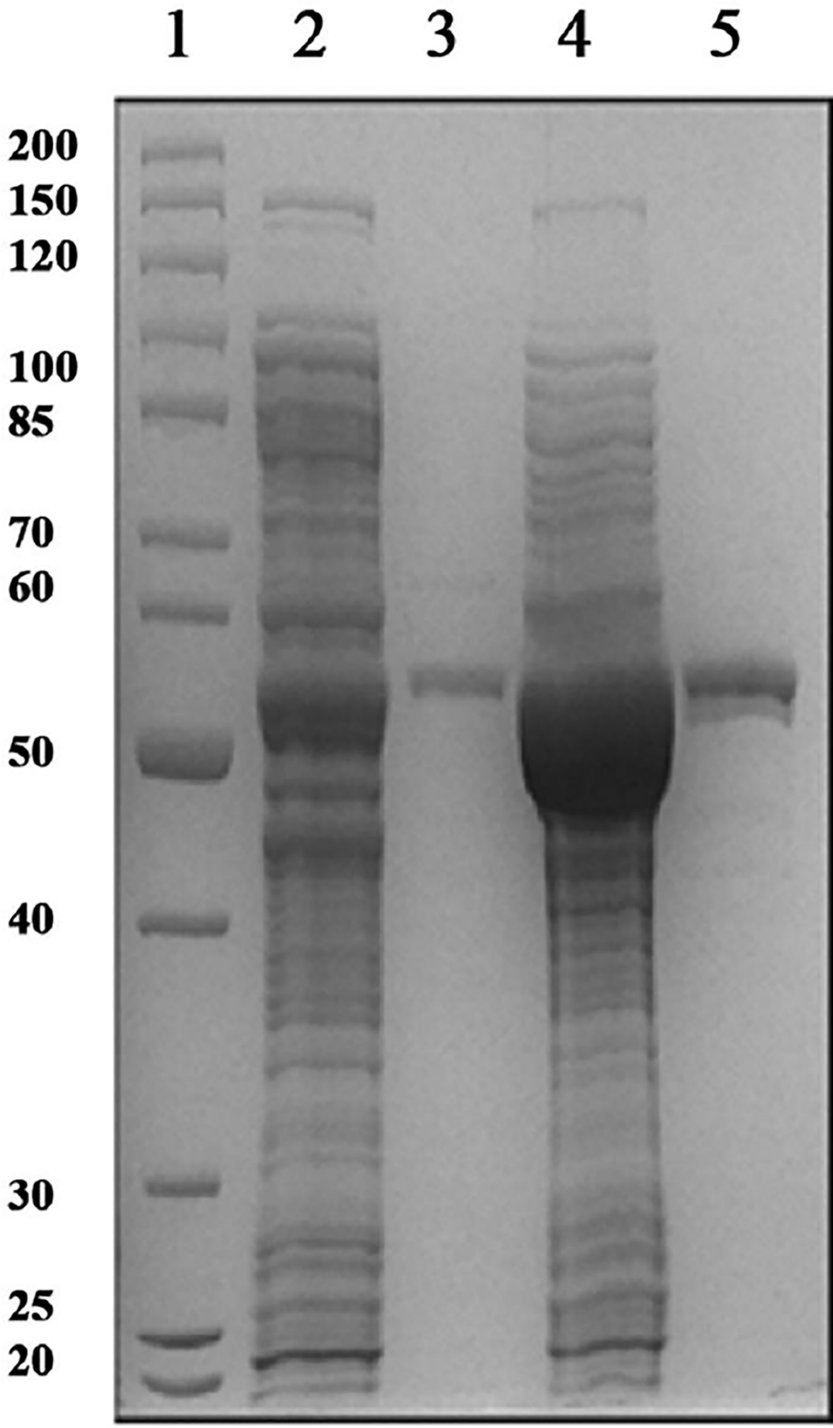
12% SDS-PAGE gel of cell-free *E*. *coli* extracts, as well as purified adenylylated and deadenylylated *Mtb*GS. Lane 1: molecular weight marker with size in kDa indicated on left. Lane 2: Adenylylated *Mtb*GS cell-free extract. Lane 3: Adenylylated *Mtb*GS purified protein. Lane 4: Deadenylylated *Mtb*GS cell-free extract. Lane 5: Deadenylylated *Mtb*GS purified protein.

Deadenylylated *Mtb*GS was expressed from pTBSK in *E*. *coli* YMC11E. Adenylylated *Mtb*GS was produced in YMC11E(DE3) via co-expression of *Mtb*GS in pTBSK and *M*. *tuberculosis* ATase in pTBGlnE. *Mtb*GS was expressed constitutively, while ATase production was induced by the addition of IPTG. The two plasmids used contain compatible origins of replication and can thus be maintained stably in the same strain. Fig 1 illustrates a SDS-PAGE of the cell-free *E*. *coli* soluble extracts, as well as the purified adenylylated and deadenylylated forms of *Mtb*GS. The average protein concentrations from three purifications of adenylylated and deadenylylated *Mtb*GS are 120 ± 28 and 145 ± 49 μg.ml^-1^, respectively. The estimated size is in agreement with the theoretical ~53.4 kDa per subunit of the *Mtb*GS dodecamer.

### Determination of the adenylylation state of adenylylated and deadenylylated *Mtb*GS

**Fig 2:**
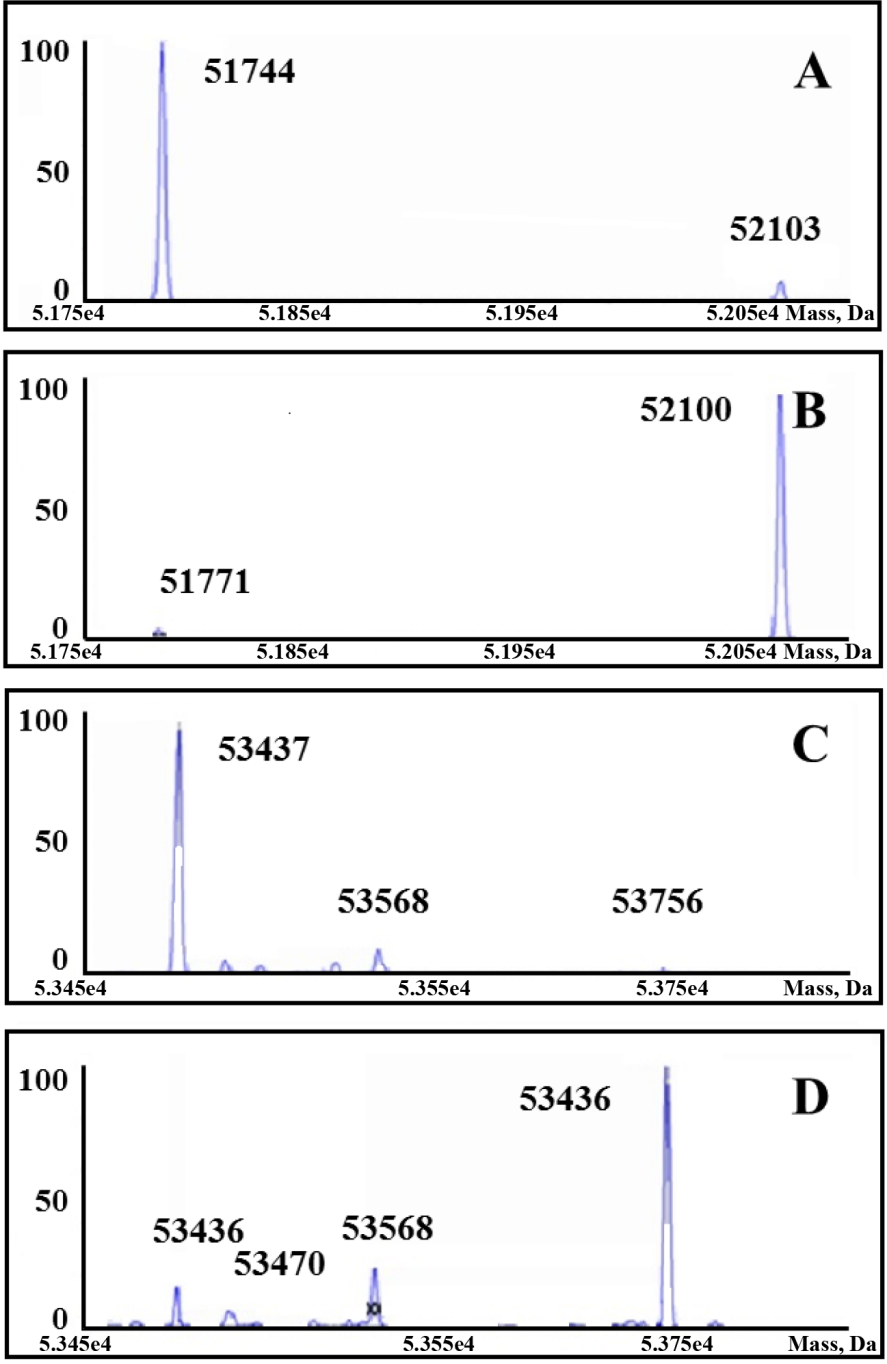
Mass spectra of *M*. *tuberculosis* and *E*. *coli* GS purified proteins. The calculated mass and presence of the major peak is shown within each spectrum (A) deadenylylated *E*. *coli* GS, (B) adenylylated *E*. *coli* GS, (C) deadenylylated *M*. *tuberculosis* GS, (D) adenylylated *M*. *tuberculosis* GS.

MS analysis of deadenylylated *Mtb*GS, yielded a single peak with a calculated mass of 53,438.00 Da (Fig 2), in agreement with the theoretical mass of 53.4 kDa. Deadenylylated *E*. *coli* GS had a calculated mass of 51,773.00 Da (theoretical mass = 51.7 kDa). Adenylylated *E*. *coli* GS was calculated to be 52,102.00 Da (theoretical mass = 52.1 kDa), with a single peak indicating 100% adenylylation. The 330 Da adenyl moiety accounts for the mass difference between the adenylylated and deadenylylated forms of each enzyme. Adenylylated *Mtb*GS would not ionise properly, therefore the spectra obtained (Fig 2) that shows 85% adenylylation, could therefore be potentially higher than 85%. In order to confirm adenylylation of *Mtb*GS, other methods were also pursued.

**Fig 3:**
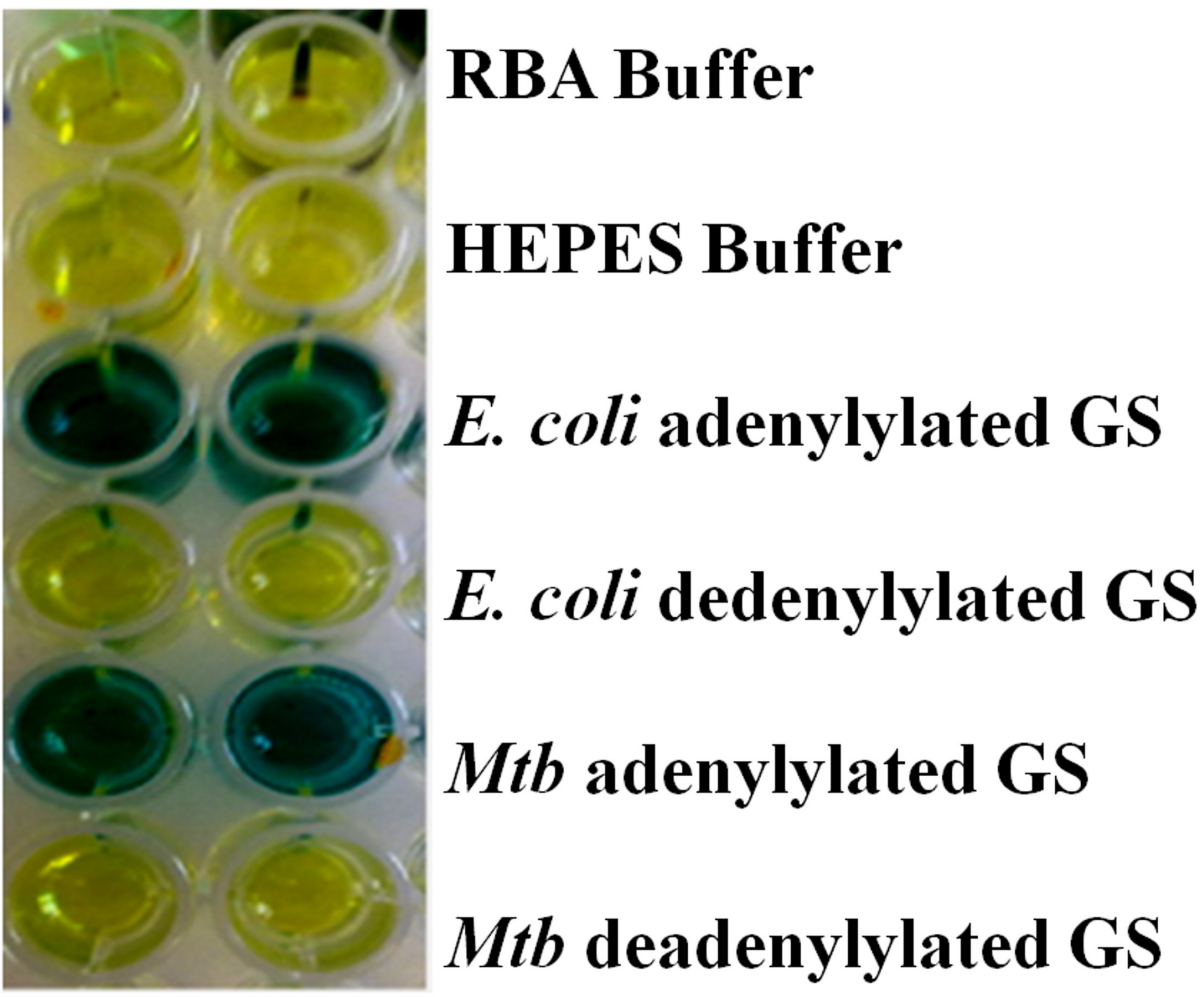
Hydrolysis of *M*. *tuberculosis* and *E*. *coli* GS purified protein (adenylylated and deadenylylated) for phosphate concentration determination. A blue colour indicates the presence of free phosphate.

The hydrolysis of GS for determination of phosphate content was carried out on the purified enzymes to confirm the degree of adenylylation (Fig 3). Each adenyl moiety contains 1 phosphate, and 1 μM of GS (containing 12 subunits) should contain 12 μM of phosphate, if each subunit is adenylylated. The result obtained for the adenylylated *Mtb*GS enzyme was the formation of 0.93 μM phosphate produced per μM GS active site, *i*.*e*. 93% adenylylated. In the case of the deadenylylated enzyme there was no formation of phosphate. The results obtained for *E*. *coli* GS were 0.95 μM and 0 μM for adenylylated and deadenylylated GS, respectively.

The γ-glutamyl transferase enzyme assay was carried out with purified GS to: i) confirm the functionality of the purified *Mtb*GS enzymes, and ii) assess the degree of adenylylation of the purified enzyme. At a specific pH, the total enzyme activity of GS (both adenylylated and deadenylylated) occurs in the presence of Mn^2+^. At the same pH in the presence of Mn^2+^ and an excess Mg^2+^, only the deadenylylated component of the enzyme activity is measured. The resultant ratio is then used to calculate the degree of adenylylation of the enzyme. After three purifications, the typical degree of adenylylation for deadenylylated *Mtb*GS is 3 ± 2%, while the adenylylated form is 68 ± 4% adenylylated. For comparison purposes, adenylylated and deadenylylated *E*. *coli* GS yielded results of 0% and 87 ± 3% adenylylation, respectively.

A summary of the adenylylation state of the *M*. *tuberculosis* GS and *E*. *coli* GS as determined by the different analysis techniques is out lined in Table 1.

**Table 1:** Summary of the adenylylation states of *M*. *tuberculosis* and *E*. *coli* GS determined by different methods. (1) mass spectroscopy, (2) hydrolysis and phosphate concentration determination and (3) γ-glutamyl transferase assay.

	*M*. *tuberculosis*		*E*. *coli*	
	Deadenylylated	Adenylylated	Deadenylylated	Adenylylated
MS	0%	85%	7%	100%
Phosphate Concentration	0 μM	0.93 μM	0 μM	0.95 μM
γ-glutamyl transferase	3 ± 2%	68 ± 4%	0	87 ± 3%

### Specific activity, inhibitor studies, dose-response assays and *M*.*tb* BACTEC 460TB™ assays

The specific activity of the adenylylated and deadenylylated *Mtb*GS were determined. The conversion of ATP to ADP was measured by HPLC and the typical specific activity of adenylylated and deadenylylated forms of *M*. *tuberculosis* GS are 0.010 and 0.015 μmol ADP/min/mg protein, respectively.

**Fig 4:**
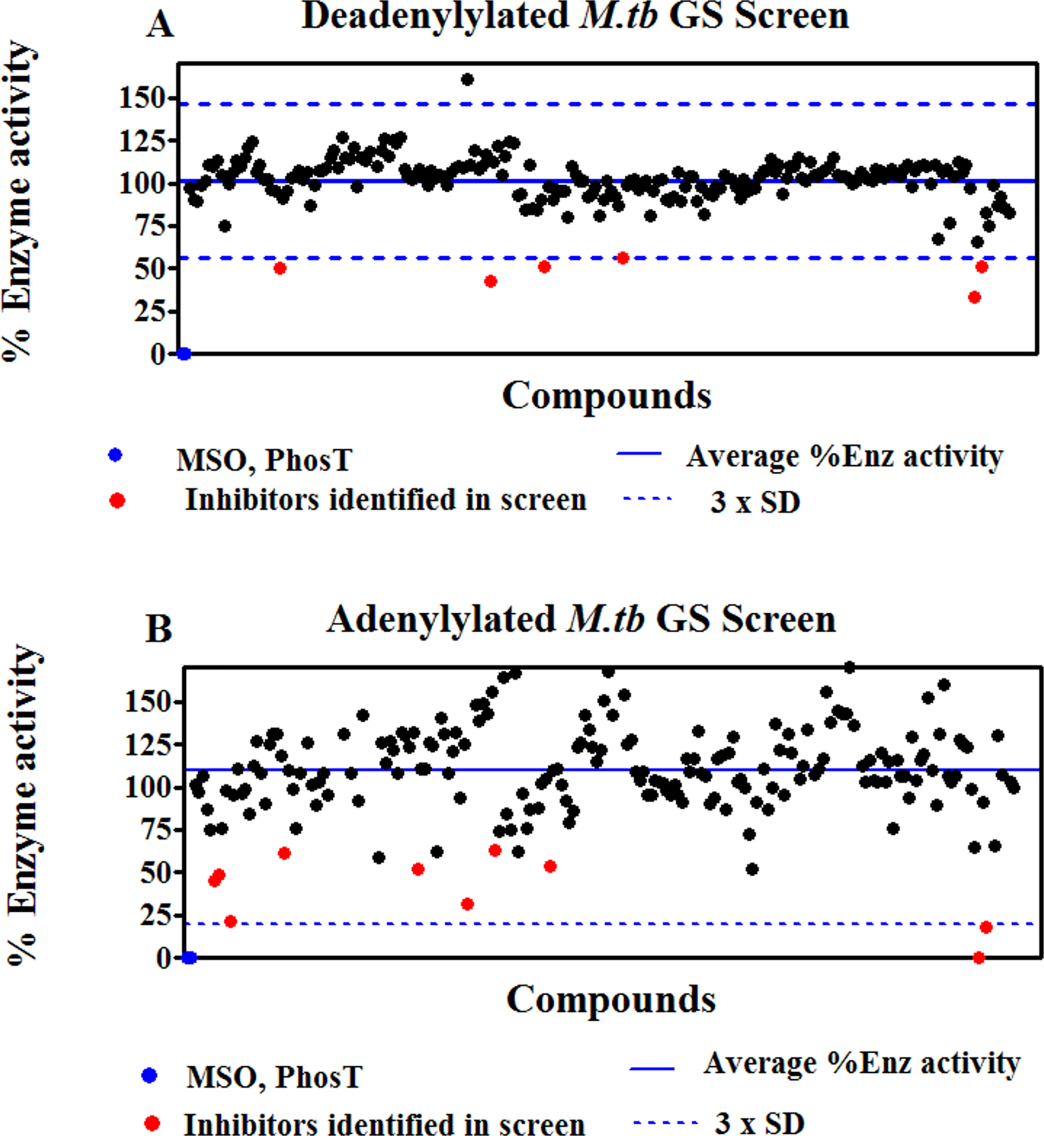
*M*. *tuberculosis* GS enzyme screen using the pre-incubation protocol. The residual percentage adenylated (B) and deadenylated (A) GS enzyme activities after incubation with 10μM of the individual compounds are shown, along with horizontal lines depicting the average % enzyme activity obtained with all compounds, as well as an indication of the confidence interval of the results, expressed as the average activity ± 3 x standard deviation (SD). Percentage activity obtained with methionine sulfoximine (MSO) and phosphinothricin (PhosT) is shown in blue. Candidate inhibitors previously identified in the adenylated GS screen are shown as red datapoints. Additional compounds selected for further evaluation are shown as orange datapoints.

Inhibitor studies with a library of 213 ATP scaffold based inhibitors were carried out to reflect the differential inhibition of adenylated and deadenylated *Mtb*GS. These assays were also performed using the pre-incubation protocol, where the inhibitors were allowed to interact with either adenylated or deadenylated *Mtb*GS before the addition of substrates sodium glutamate and ATP. Based on the results illustrated with the deadenylylated enzyme in Fig 4A, six candidate inhibitors (indicated by the red data points) showed promising inhibitory activities. With the adenylylated enzyme, the % enzyme activities in the presence of the individual compounds varied widely, producing a pronounced scatter of values around the mean and consequently a very wide confidence interval (Fig 4B) nevertheless ten inhibitors (indicated by the red data points) showed promising inhibitory activities with one inhibitor yielding an inhibition of 99%. The scatter associated with the data when using the adenylylated GS is probably associated with inter subunit allosteric regulation of adenylylated GS [42]. Some compounds therefore allosterically activate the enzyme.

**Fig 5:**
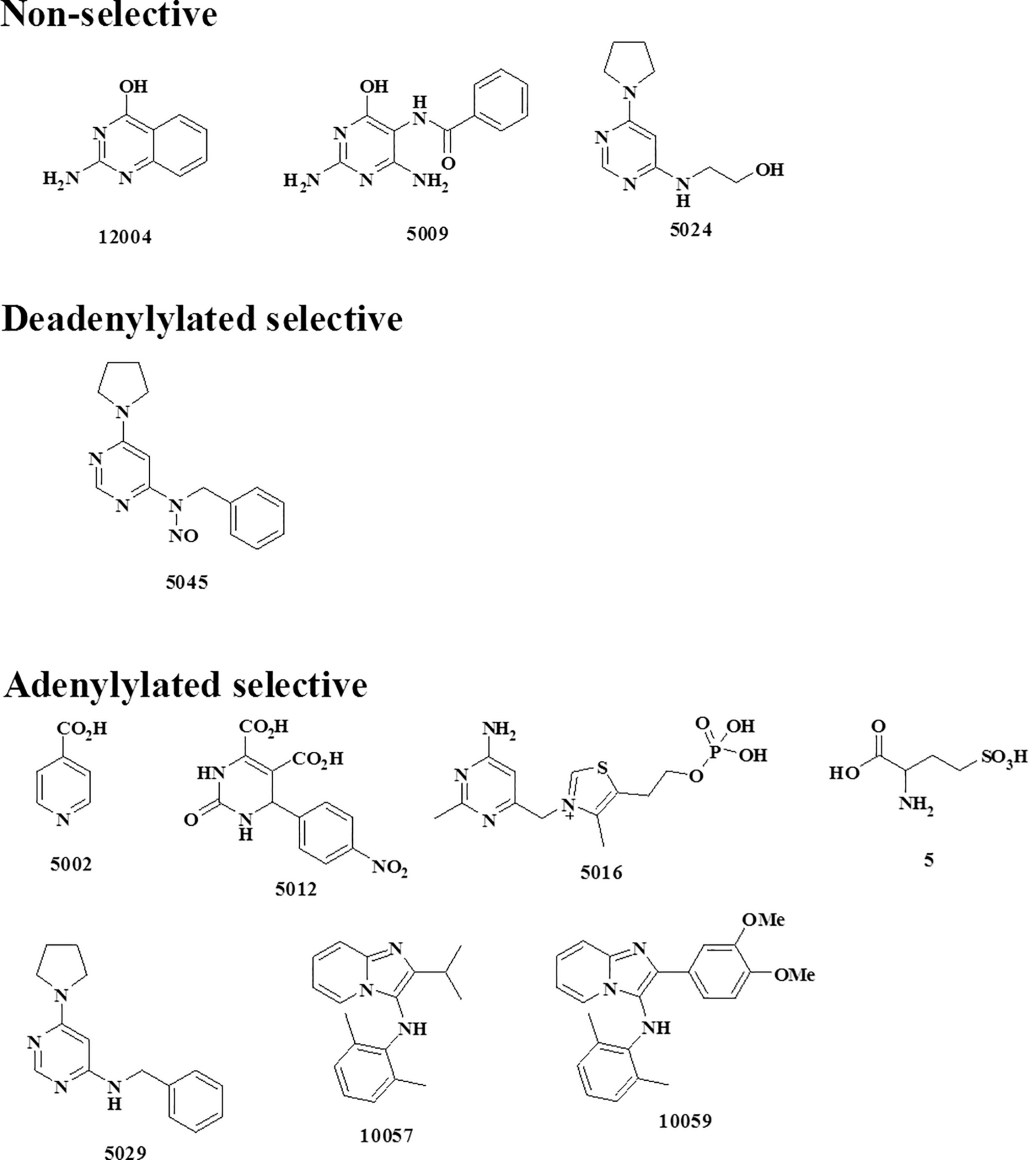
Chemical structures of the 11 synthesized ATP scaffold based inhibitors which shown promising inhibition of *M*. *tuberculosis* GS enzymes. They are divided into three distinct groups (1) non-selective (2) Deadenylylated selective and (3) Adenylylated selective.

Fixed concentration evaluation of eleven candidate inhibitors were tested on both adenylated and deadenylated *Mtb*GS. Based on the results indicated in Table 2 there is a clear indication of differential inhibition between the two forms of *Mtb*GS. One compound (5045) displayed significant selectivity for the deadenylylated *Mtb*GS, while seven compounds suggested specificity for the adenylylated *Mtb*GS and three appeared relatively non-selective. The chemical structures of these candidate inhibitors are indicated in Fig 5.

**Table 2:** Fixed concentration evaluation of 11 candidate inhibitors in *M*.*tuberculosis* GS enzyme assays using the pre-incubation protocol. The percentage enzyme inhibition obtained with the compounds (10μM final concentration) in 4 separate screens is shown, along with the average inhibition for each compound over the 4 screens and the corresponding standard deviation (SD). The colour coding of the highlighted table cells is clarified in the bottom legend. Compounds were defined as selective for the adenylylated or deadenylylated form of the enzyme based on at least a 30% difference in inhibition.

	Adenylated *Mtb* GS (% inhibition)						Deadenylated *Mtb* GS (% inhibition)					
Compound (10μM)	Screen 1	Screen 2	Screen 3	Screen 4	AVG	SD	Screen 1	Screen 2	Screen 3	Screen 4	AVG	SD
PhosT	100	95	97	100	98	2	100	98	100	100	100	1
MSO	100	100	96	100	99	2	100	100	99	100	100	1
5	48	33	40	29	38	8	0	0	6	0	2	3
5002	68	40	38	35	45	15	0	10	8	14	8	6
5009	37	50	50	22	40	13	57	41	44	44	47	7
5012	55	31	32	42	40	11	14	16	4	24	15	8
5016	51	43	46	30	43	9	7	19	3	13	11	7
5024	46	48	43	36	43	5	49	44	52	44	47	4
5029	79	46	56	60	60	14	5	8	7	9	7	2
5045	0	0	0	0	0	0	44	45	54	48	48	5
10057	100	99	98	100	99	1	67	62	47	58	59	9
10059	82	99	82	100	91	10	49	48	36	43	44	6
12004	39	32	33	30	34	4	50	43	38	38	42	6
		Non-selective										
		Adenylylated										
		Deadenylylated										

**Fig 6:**
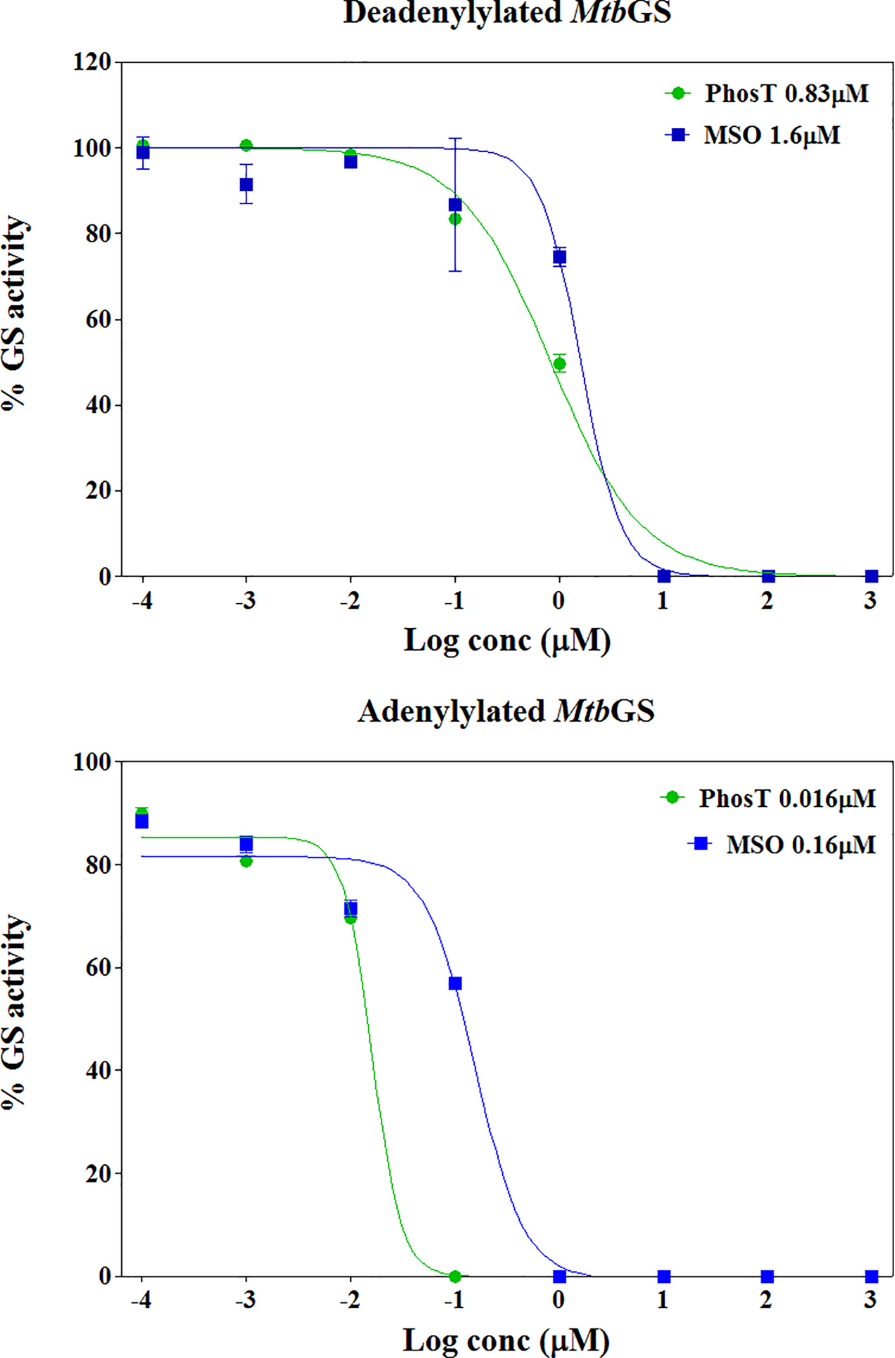
Dose-response assays for MSO and PhosT standard inhibitors. **Percentage GS activity was plotted against log (compound concentration) and sigmoidal dose-response curves fitted to the data points using non-linear regression analysis.** The curves were used to derive the IC_50_ values for the inhibitors (displayed in the top right legend).

Dose-response assays were carried out to obtain a more accurate reflection of the activity of adenylated and deadenylated *Mtb*GS. These assays were performed on the 2 forms of the enzyme to derive compound IC_50_ (50% inhibitory concentration) values, using the standard GS inhibitors MSO and PhosT. Dose-response assays were performed using a pre-incubation protocol, allowing the inhibitor to interact with the enzyme before addition of substrates sodium glutamate and ATP. Based on the results illustrated in Fig 6, IC_50_ values derived from the dose-response curves suggested, firstly, that adenylylated *Mtb*GS is more sensitive to inhibition by the standard inhibitors than the deadenylylated form of the enzyme and, secondly, that PhosT is a more potent inhibitor of the *Mtb* enzymes than MSO. Compounds 10057 and 10059 both gave IC_50_ concentrations less than 100 μM. In duplicate assays run on different days in triplicate the IC_50_ concentrations obtained were 3.51 and 15.6 μM and 13.2 and 17.1 μM, for the adenylylated enzyme and deadenylylated enzyme, respectively.

*M*.*tb* BACTEC 460TB™ assays were carried out on compounds identified following the primary screening for enzymatic activity against adenylylated and deadenylylated *M*.*tb* GS. Compounds identified (see Fig 5) were tested for activity against *M*.*tb* H37Rv reference strain for antibacterial activity. Growth inhibition was calculated as a percentage of the growth index (GI) at day 6 of incubation where the GI of 1.0% of the control culture equalled 50 GI units. Of the compounds directed against adenylylated GS (Fig 5), compound 10057 showed 53% (+/- 12.7) growth inhibition at 100 μM and also compound 5029 gave 66% (+/- 2.8) inhibition at 100uM against H37Rv on day 6 of incubation relative to the control cultures. However, the growth rate (ΔGI = GI at day+1 minus GI at day) in both cases continuously increased over the period of incubation and past day 6 of incubation indicating that bacterial doubling is taking place although at a slower rate. Most the compounds (5024, 12004, 5009, 5012, 5002, and 5016) showed no activity against *M*.*tb* H37Rv at 100 μM final concentration. Compound 5045, designed to be directed against the de-adenylylated GS (Fig 5) showed an inhibition of 98% (+/- 8.8) growth of the H37Rv mycobacterial cultures.

**Fig 7:**
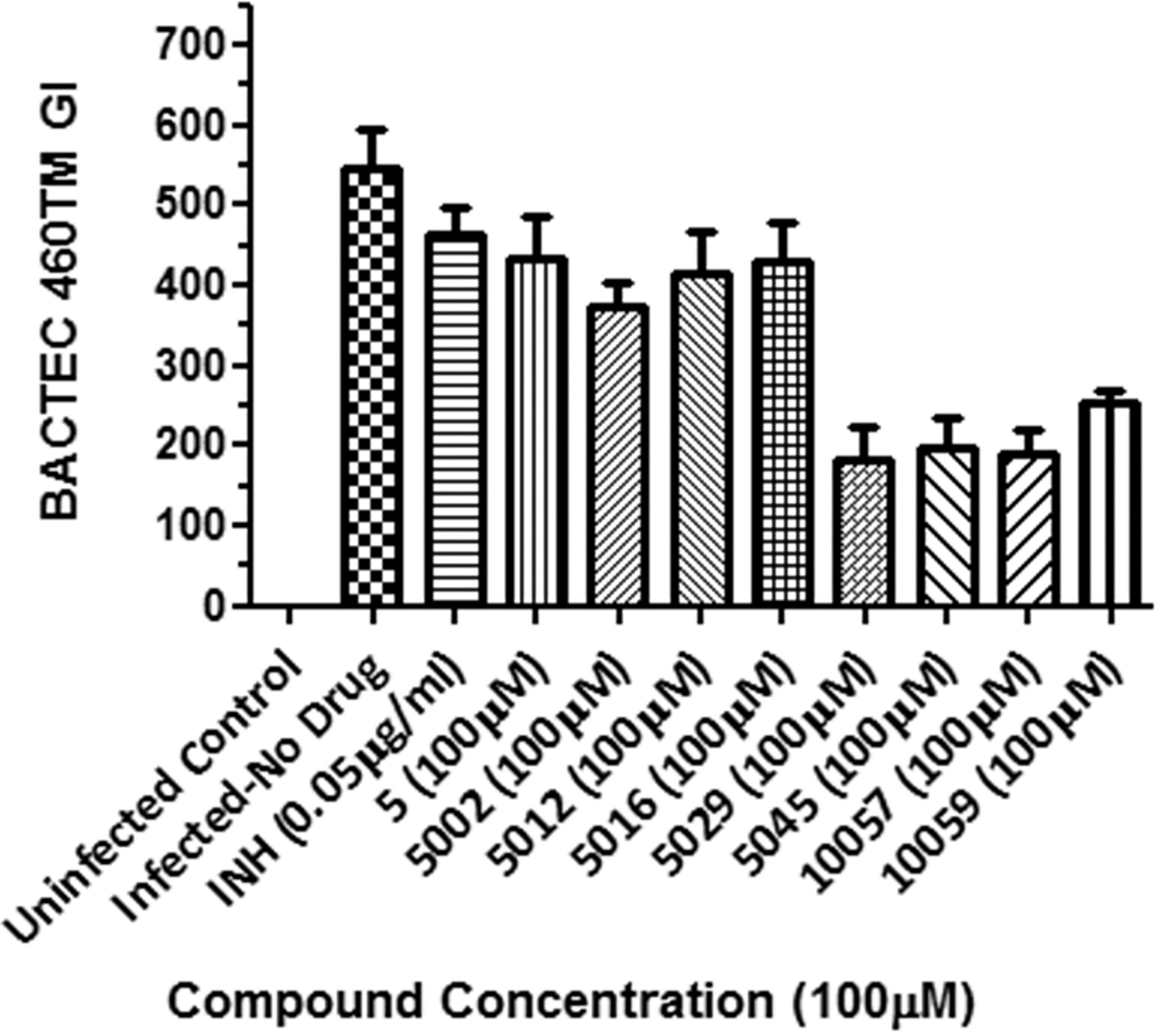
Intracellular survival of *M*.*tb* (Beijing220) in mouse bone-marrow derived macrophages after 4-day post-infection period followed by intervention on D4 PI with different compounds, MOI 2:1, 4 day incubation at 37°C, 5% CO_2_. Cells were sacrificed on Day 2 Post Drug Intervention. Only Drugs 5029, 5045, 10057 and 10059 showed moderate activity. Where not shown, error bars fall within symbols.

The 8 selective compounds identified in the enzyme assays namely compounds that were de-adenylylated selective (5, 5002, 5012, 5016, 5029, 10057, 10059) and deadenylylated selective (5045), were tested in mouse bone-marrow derived macrophages infected with a clinical multidrug-resistant strain actively spreading in the Western Cape region of South Africa (Johnson *et*. *al*., 2010; Leisching *et*. *al*., 2016). Only compounds 5029, 5045, 10057 and 10059 showed activity at a concentration of 100 μM (see Fig 7). The percentage killing exhibited by compounds 5029, 5045, 10057 and 10059 were 67%, 64%, 65% and 54% respectively at 100 μM.

**Fig 8:**
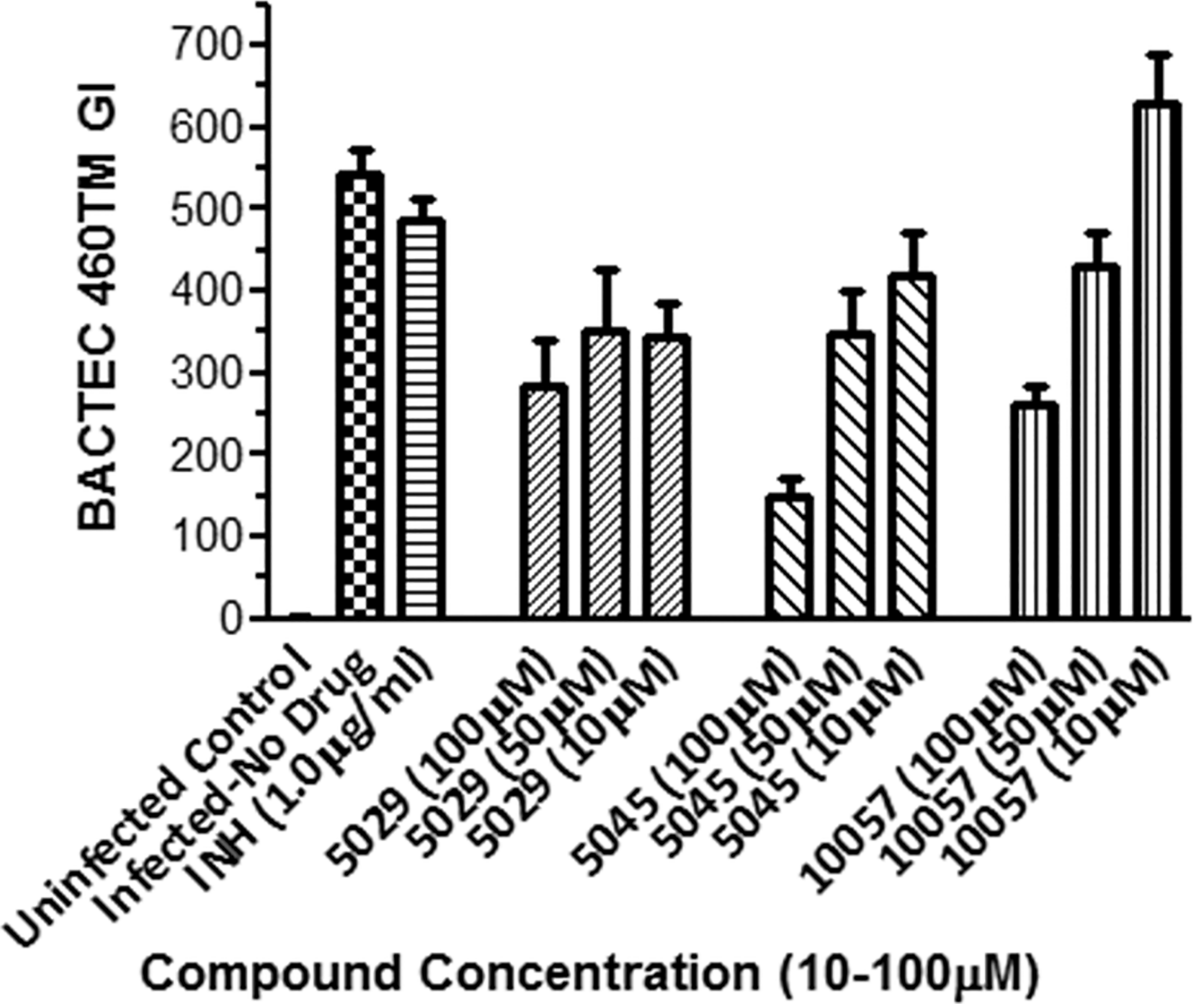
Intracellular survival of *M*.*tb* (Beijing220) in mouse bone-marrow derived macrophages after 4-day post-infection period followed by intervention on D4 PI with different compounds, MOI 2:1, 4 day incubation at 37°C, 5% CO_2_. **Cells were sacrificed on Day 2 Post Drug Intervention.** Drug 5045 showed the best activity (73% killing) at 100 μM. Where not shown, error bars fall within symbols.

The most active compounds 5029, 5045 and 10057 were tested further in macrophages at concentrations 10 μM, 50 μM and 100 μM for 2 days (Fig 8). In this experiment compound 5045 showed the best activity (73% killing) at 100 μM. The growth inhibitory effect of compound 5045 correlated well with the high inhibition observed in the extracellular BACTEC assay against *M*.*tb* H37Rv (98% inhibition at 100μM).

## Discussion

*M*. *tuberculosis* GS is a potentially valuable therapeutic target for TB drug intervention. Its regulation via adenylylation of a tyrosine residue on each subunit makes it distinct from the human form of the enzyme. Prokaryotic GS is regulated via a complex cascade that is based on the availability of NH_4_^+^ and glucose to the organism and the intracellular concentrations of 2-ketoglutarate and glutamine [1, 4–8]. This regulation results in the adenylylation or deadenylylation of the GS with a concomitant switch in the enzymes affinity from Mn^2+^ to Mg^2+^[1, 4–8, 30]. The coordination chemistry of Mn^2+^ to Mg^2+^ are significantly different enough to change the shape of the active site of the GS as is the effect of adenylylation. No crystal structure exits for the fully adenylylated form of the enzyme [31]. A number of studies have been undertaken targeting *Mtb*GS as a potential therapeutic target however, in none of these investigations was an attempt made to exploit this dichotomy that exists between adenylylated and deadenylylated GS [32–38]. Previous reports of heterologous expression of *Mtb*GS in *E*. *coli* have shown that the endogenous ATase activity of *E*. *coli* does not adenylylate *Mtb*GS sufficiently, with only 25% of the *Mtb*GS subunits produced displaying adenylylation [39]. The use of this expression system was therefore not considered optimal for the expression of adenylylated *Mtb*GS for further study.

Here, we have described an *E*. *coli* production system, lacking endogenous GS and ATase activity, which utilises the co-expression of the *M*. *tuberculosis* ATase with *Mtb*GS. Each gene was provided on a separate plasmid, the *glnA* gene on a pBluescript SKII^+^ plasmid with the ColE1 origin of replication, and the *glnE* gene on a pCDFDuet-1 plasmid containing a CloDF13 replicon. These replicons are compatible, and the two plasmids can be stably co-maintained, provided the relevant antibiotic selective pressure is exerted: ampicillin for pBluescript SKII^+^ and streptomycin for pCDFDuet-1[40]. In this way, we have produced *Mtb*GS that has a better adenylylation state than any previously reported. Three methods were used to assess the adenylylation of *Mtb*GS, and *E*. *coli* adenylylated and deadenylylated GS. The *E*. *coli* enzymes were produced recombinantly from pBluescript SKII^+^ in *E*. *coli* production strains lacking endogenous GS (for deadenylylated enzyme) or both GS and uridylyl transferase (adenylylated enzyme). Table 1 gives a summary of the results obtained. MS spectra showed distinct peaks for adenylylated and deadenylylated enzymes, with calculated masses agreeing with the theoretical values. Based on this data, it can be concluded that the adenylylation state of adenylylated *Mtb*GS expressed in this novel system is at least 85% based on the MS data, but possibly up to 93% based on phosphate hydrolysis. Based on this data this investigation was carried out to demonstrate the proof-of-concept that compounds could be obtained that selectively inhibit either adenylylated or deadenylylated *MtbGS* an that these compounds would inhibit *Mtb* in macrophages.

The fixed concentration inhibition assays of the 213 ATP based scaffold inhibitors produced 11 potential inhibitors. The scatter associated with the data when using the adenylylated GS is probably associated with inter subunit allosteric regulation of adenylylated GS [42]. Some compounds therefore allosterically activate the enzyme. This would be achieved as a result of allosteric regulation occurring between two GS subunits as a result of the adenylylation. It is therefore conceivable that inhibitors binding one GS subunit may either activate or inhibit the binding of another inhibitor or ATP to the associated allosteric site. There was also differential inhibition of the adenylylated and deadenylylated *Mtb*GS. Seven of the potential inhibitors were directed against adenylylated *Mtb*GS and one was directed against deadenylylated *Mtb*GS. Two of the inhibitors namely 10057 and 10059 showed promising anti-*Mtb*GS activity against adenylylated *Mtb*GS with 99% and 91% inhibition respectively. The IC_50_ concentrations obtained for compounds 10057 and 10059 were approximately 9.6 μM and 15 μM, for the adenylylated enzyme and deadenylylated enzyme, respectively. Taking into account the large variation in ATP and glutamine concentrations used in the screening assays similar low μM IC_50_ concentrations were obtained in the enzyme screens [32–38]. Imidazopyridine amides have been found to inhibit *Mtb* in other studies indicating an alternative mechanism via the cytochrome cytochrome *bc*_1_ complex impacting on the homeostasis of ATP synthesis [39]. The inhibition of glutamine synthetase may also impact the ATP homeostasis as the resultant accumulation of α-ketoglutarate, as a result of inhibiting glutamine synthetase, may lead to the slowing down of metabolic flux via the TCA cycle.

Compounds 10057 and 10059 are structurally very similar. Also in the macrophage assays compounds 10057, 10059 and 5029 which were GS-adenylylated specific, showed comparable activities in the BACTEC assays. This indicates that these compounds can inhibit *M*.*tb* as effectively in a macrophage environment. However, compound 5045, specific to the de-adenylylated form of GS, inhibited *M*.*tb* killing in both the in vitro and ex vivo macrophage models effectively at 98% and 73% respectively at 100μM. The GS adenylylation/deadenylylation status of the *M*.*tb* strains used in the BACTEC assay and the macrophage assays were not known which may explain the high killing effect of both the adenylylation- and de-adenylylation specific compounds. In further development, attention may be paid to improving the selectivity of the compounds *vis-à-vis* mammalian forms of GS, to reduce the possibility of side-effects.

## Supporting information

S1 File Preparation of adenylylated and deadenylylated *E*. *coli* GS. (DOCX)

## References

[1] Shapiro BM, Stadman ER. The regulation of glutamine synthesis in microorganisms. Ann Rev Microbiol. 1970;24: 501–524. 4927139 10.1146/annurev.mi.24.100170.002441

[2] Kumada Y, Benson DR, Hillemann TJ, Hosted DA, Rochefort CJ, Thompson W, et al Evolution of the glutamine synthetase gene, one of the oldest existing and functioning genes. Proc Natl Acad Sci USA. 1993;90: 3009–3013. 8096645 10.1073/pnas.90.7.3009 PMC46226

[3] Brown JR, Masuchi Y, Robb FT, Doolittle WF. Evolutionary relationships of bacterial and archaeal glutamine synthetase genes. J Mol Evol. 1994;38: 566–576. 7916055 10.1007/BF00175876

[4] Tyler B. Regulation of the assimilation of nitrogen compounds. Ann Rev Biochem. 1978;47: 1127–1162. doi: 10.1146/annurev.bi.47.070178.005403 28074 10.1146/annurev.bi.47.070178.005403

[5] Gaillardin CM, Magasanik B. Involvement of the product of the *glnF* gene in the autogenous regulation of glutamine synthetase formation in *Klebsiella aerogenes*. J Bacteriol. 1978;133: 1329–1338. 25264 10.1128/jb.133.3.1329-1338.1978 PMC222170

[6] Foor F, Janssen KA, Magasanik B. Regulation of synthesis of glutamine synthetase by adenylylated glutamine synthetase. Proc Natl Acad Sci USA. 1975;72: 4844–4848. 1744 10.1073/pnas.72.12.4844 PMC388828

[7] Janssen KA, Magasanik B. Glutamine synthetase of *Klebsiella aerogenes*: genetic and physiological properties of mutants in the adenylylation system. J Bacteriol. 1977;129: 993–1000. 14117 10.1128/jb.129.2.993-1000.1977 PMC235039

[8] Senior PJ. Regulation of nitrogen metabolism in *Escherichia coli* and *Klebsiella aerogenes*: studies with the continuous-culture technique. J Bacteriol. 1975;123: 407–418. 238954 10.1128/jb.123.2.407-418.1975 PMC235743

[9] Ginsberg A, Stadtman ER. Regulation of glutamine synthetase in *Escherichia coli* In: Prusiner SR, Stadman ER, editors. Enzymes of Glutamine Metabolism, Academic Press, New York, 1973; p. 9–44.

[10] Wolhueter RM, Schutt H, Holzer H. Regulation of glutamine synthetase *in vivo* in *E*. *coli* In: Prusiner SR, Stadman ER, editors. Enzymes of Glutamine Metabolism, Academic Press, New York, 1973; p. 45–61.

[11] Bender RA, Janssen KA Resnick AD, Blumenberg M, Foor F, Magasanik B. Biochemical parameters of glutamine synthetase from *Klebsiella aerogenes*. J Bacteriol. 1977;129: 1001–1009. 14104 10.1128/jb.129.2.1001-1009.1977 PMC235040

[12] Bloom FR, Streicher SL, Tyler B. Regulation of enzyme synthesis by glutamine synthetase of *Salmonella typhimurium*: a factor in addition to glutamine synthetase is required for activation of enzyme formation. J Bacteriol. 1977;130: 983–990. 16868 10.1128/jb.130.3.983-990.1977 PMC235318

[13] Holzer H, Schutt H, Mašek Z, Mecke D. Regulation of two forms of glutamine synthetase in *Escherichia coli* by the ammonium content of the growth medium. Proc Natl Acad Sci USA. 1968;60: 721–724. 4885513 10.1073/pnas.60.2.721 PMC225105

[14] Woolfolk CA, Shapiro B, Stadtman ER. Regulation of glutamine synthetase I. Purification and properties of glutamine synthetase from *Escherichia coli*. Arch Biochem Biophys. 1966;116: 177–192. 5336023 10.1016/0003-9861(66)90026-9

[15] Kustu SG, McKereghan K. Mutations affecting glutamine synthetase activity in *Salmonella typhimurium*. J Bacteriol. 1975;122: 1006–1016. 238935 10.1128/jb.122.3.1006-1016.1975 PMC246153

[16] Shapiro BM, Kingdon HS, Stadtman ER. Regulation of glutamine synthetase. VII. Adenylyl glutamine synthetase: a new form of the enzyme with altered regulatory and kinetic properties. Proc Natl Acad Sci USA. 1967;58: 642–649. 4860756 10.1073/pnas.58.2.642 PMC335683

[17] Shapiro BM, Stadtman ER. 5'-adenylyl-*O*-tyrosine. The novel phosphodiester residue of adenylylated glutamine synthetase from *Escherichia coli*. J Bio. Chem. 1968;243: 3769–3771. 4298074

[18] Kingdon HS, Shapiro BM, Stadtman ER. Regulation of glutamine synthetase. VIII. ATP: glutamine synthetase adenylyltransferase, an enzyme that catalyzes alterations in the regulatory properties of glutamine synthetase. Proc Natl Acad Sci USA. 1967;58: 1703–1710. 4867671 10.1073/pnas.58.4.1703 PMC223983

[19] Mecke D, Wulff K, Liess K, Holzer H. Characterization of a glutamine synthetase inactivating enzyme from *Escherichia coli*. Biochem Biophys Res Commun. 1966;24: 452–458. 5338440 10.1016/0006-291x(66)90182-3

[20] Reitzer LJ, Magasanik B. Ammonia assimilation and the biosynthesis of glutamine, glutamate, aspartate, asparagine, l-alanine, and d-alanine In: Neidart FC, Ingarhou JLL, Low KB, Magasanik B, Schaechter M Nunberger HE, editors. *Escherichia coli* and *Salmonella typhimurium*. Cellular and molecular biology, American Society for Microbiology, Washington DC, 1987; p. 302–320.

[21] Shapiro BM, Ginsburg A. Effects of specific divalent cations on some physical and chemical properties of glutamine synthetase from *Escherichia coli*. Taut and relaxed enzyme forms. Biochemistry. 1968;7: 2153–2167. 4873174 10.1021/bi00846a018

[22] Reynaud C, Etienne G, Payron P, Lenelle MA, Daffe M. Extracellular enzyme activities potentially involved in the pathogeniticty of *Mycobacterium tuberculosis*. Microbiol. 1998;144: 577–587. 10.1099/00221287-144-2-577 9493394

[23] Harth G, Horwitz MA. An inhibitor of exported *Mycobacterium tuberculosis* glutamine synthetase selectively blocks the growth of pathogenic mycobacteria in axenic culture and in human monocytes: extracellular proteins as potential drug targets. J Exp Med. 1999;189: 1425–1435. 10224282 10.1084/jem.189.9.1425 PMC2193054

[24] Harth G, Horwitz MA. Inhibition of *Mycobacterium tuberculosis* glutamine synthetase as a novel antiobiotic strategy against tuberculosis: demonstration of efficacy *in vivo*. Infect Immune. 2003;71: 456–464. 10.1128/IAI.71.1.456-464.2003 PMC143262 12496196

[25] Tullius MV, Harth G, Horwitz MA. Glutamine synthetase GlnA1 is essential for growth of *Mycobacterium tuberculosis* in human THP-1 macrophages and guinea pigs. Infect Immun. 2003;71: 3927–3936. doi: 10.1128/IAI.71.7.3927-3936.2003 12819079 10.1128/IAI.71.7.3927-3936.2003 PMC162033

[26] Wietzerbin J, Lederer F, Petit JF. Structural study of the poly-l-glutamic acid of the cell wall of *Mycobacterium tuberculosis* var *hominis*, strain Brevannes. Biochem Biophys Res Commun. 1975;62: 246–252. 803372 10.1016/s0006-291x(75)80130-6

[27] Hirschfield GR, McNeil M, Brennan PJ. Peptidoglycan-associated polypeptides of *Mycobacterium tuberculosis*. J Bacteriol. 1990;172: 1005–1013. 2105289 10.1128/jb.172.2.1005-1013.1990 PMC208529

[28] Harth G, Horwitz MA. Expression and efficient transport of enzymatically active *Mycobacterium tuberculosis* glutamine synthetase in *Mycobacterium smegmatis* and evidence that information for export is contained within the protein. J Biol Chem. 1997;272: 22728–22735. 9278431 10.1074/jbc.272.36.22728

[29] Singh J, Joshi C, Bhatnagar R. Cloning and expression of mycobacterial glutamine synthetase gene in *Escherichia coli*. Biochem Biophys Res Comun. 2004;317:634–638. 10.1016/j.bbrc.2004.03.094 15063805

[30] Mehta R, Pearson JT, Mahajan S, Nath A, Hickey MJ, Sherman DR, et al Adenylylation and catalytic properties of *Mycobacterium tuberculosis* glutamine synthetase expressed in *Escherichia coli* versus Mycobacteria. J Biol Chem. 2004:279: 22477–22482. doi: 10.1074/jbc.M401652200 15037612 10.1074/jbc.M401652200

[31] (2017.07.24) http://www.rcsb.org/pdb/home/home.do

[32] Lagerlund O, Odell LR, Mowbray SL, Nilsson MT, Krajewski WW, Nordqvist A, et al Microwave-enhanced alpha-acrylation of a protected glycine in water: evaluationof 3-phenylglycine derivatives as inhibitors of the tuberculosis enzyme, glutamine synthetase. Comb Chem High Throughput Screen. 2007;10: 783–789. 18478959 10.2174/138620707783018478

[33] Nilsson MT, Krajewski W, Yellagunda S, Prabhumurthy S, Chamarahally GN, Siddanadappa C, et al Structural basis for the inhibition of *Mycobacterium tuberculosis* glutamine synthetase by novel ATP-competitive inhibitors. J Mol Biol. 2009;393: 503–513. 10.1016/j.jmb.2009.08.028 19695264

[34] Odell LR, Nilsson MT, Gising J, Lagerhund O, Muthas D, Nordqvist A, et al Functionalized 3-amino-imidazole[1,2*a*]pyridines: A novel class of drug-like *Mycobacterium tuberculosis* glutamine synthetase inhibitors. Bioorg Med Chem Lett. 2009;19: 4790–4793. doi: 10.1016/j.bmcl.2009.06.045 19560924 10.1016/j.bmcl.2009.06.045

[35] Gising J, Nilsson MT, Odell LR, Yahiaoui S, Lindh M, Iyer H, et al Trisubstituted imidazoles as *Mycobacterium tuberculosis* glutamine synthetase inhibitors. J Med Chem. 2012;55: 2894–2898. doi: 10.1021/jm201212h 22369127 10.1021/jm201212h PMC3381009

[36] Couturier C, Silve S, Morales R, Pesseque B, Liopart S, Nair A, et al Nanomolar inhibitors of *Mycobacterium tuberculosis* glutamine synthetase 1: Synthesis, biological evaluation and X-ray crystallographic studies. Bioorg Med Chem Lett. 2015;25: 1455–1459. doi: 10.1016/j.bmcl.2015.02.035 25770781 10.1016/j.bmcl.2015.02.035

[37] Mowbray SL, Kathiravan MK, Pandey AA, Odell LR. Inhibition of glutamine synthetase: A potential drug target in *Mycobacterium tuberculosis*. Molecules. 2014;19: 13161–13176. doi: 10.3390/molecules190913161 25162957 10.3390/molecules190913161 PMC6271674

[38] Kosikowska P, Bochno M, Maceginiak K, Forlani G, Kafarski P, Berlicki L. Bisphosphonic acids as effective inhibitors of *Mycobacterium tuberculosis* glutamine synthetase. J Enzyme Inhib Med Chem. 2016;31: 931–938. doi: 10.3109/14756366.2015.1070846 26235917 10.3109/14756366.2015.1070846

[39] Pethe K, Bifani P, Jang J, Kang S, Park S, Ahn S, et al Discovery of Q203, a potent clinical candidate for the treatment of tuberculosis. Nature Medicine. 2013;19: 1157–1162. doi: 10.1038/nm.3262 23913123 10.1038/nm.3262

[40] Held D, Yaeger K, Novy R. New coexpression vectors for expanded compatibilities in *E*. *coli*. Innovations. 2003;18: 406.

[41] Backman K Chen Y-M, Magasanik B. Physical and genetic characterization of the *glnA*-*glnG* region of the *Escherichia coli* chromosome. Proc Natl Acad Sci USA. 1981;78: 3743–3747. 6115384 10.1073/pnas.78.6.3743 PMC319648

[42] Shapiro BM, Stadtman ER. Glutamine Synthetase (*Escherichia* coli) In: Hirs CHW, Timasheff SN, editors. Methods in Enzymology 130, Elsevier, 1970; p. 910–922.

[43] Laemlli UK. Cleavage of structural proteins during the assembly of the head of bacteriophage T4. Nature. 1970;227: 680–685. 5432063 10.1038/227680a0

[44] Ludden PW, Burris RH. Purification and properties of nitrogenase from *Rhodospirillum rubrum*, and evidence for phosphate, ribose and an adenine-like unit covalently bound to the iron protein. Biochem J. 1978;175: 251–259. 104713 10.1042/bj1750251 PMC1186061

[45] Kenyon CP, Steyn A, Roth RL, Steenkamp PA, Nkosi TC, Oldfield LC. The role of the C8 proton of ATP in the regulation of phosphoryl transfer within kinases and synthetases. BMC Biochem. 2011;12: 36 doi: 10.1186/1471-2091-12-36 21749731 10.1186/1471-2091-12-36 PMC3145573

[46] Collins L, Franzblau SG. Microplate alamar blue assay versus BACTEC 460 system for high-throughput screening of compounds against *Mycobacterium tuberculosis* and *Mycobacterium avium*. Antimicrob Agents Chemother. 1997;41(5):1004–1009. 9145860 10.1128/aac.41.5.1004 PMC163841

[47] Johnson R, Warren RM, van der Spuy GD, Gey van Pittius NC, Theron D, Streicher EM, et al Drug-resistant tuberculosis epidemic in the Western Cape driven by a virulent Beijing genotype strain. Int J Tuberc Lung Dis. 2010; 14(1):119–121. 20003705

[48] Leisching G, Pietersen RD, Mpongoshe V, van Heerden C, van Helden P, Wiid I, et al The host response to a clinical MDR mycobacterial strain cultured in a detergent-free environment: A global transcriptomics approach. PLoS One. 2016;11(4):e0153079 doi: 10.1371/journal.pone.0153079 27055235 10.1371/journal.pone.0153079 PMC4824497

[49] Siddiqi SH, Heifets LB, Cynamon MH, Hooper NM, Laszlo A, Libonati JP, et al Rapid broth macrodilution method for determination of MICs for Mycobacterium avium isolates. J Clin Microbiol. 1993;31(9):2332–2338. 8408551 10.1128/jcm.31.9.2332-2338.1993 PMC265756

[50] George G, Mony P, Kenneth J. Comparison of the efficacies of loop-mediated isothermal amplification, fluorescence smear microscopy and culture for the diagnosis of tuberculosis. PLoS One. 2011:6(6):e21007 doi: 10.1371/journal.pone.0021007 21695047 10.1371/journal.pone.0021007 PMC3117872

[51] De Chastellier C, Lang T, Thilo L. Phagocytic processing of the macrophage endoparasite, *Mycobacterium avium*, in comparison to phagosomes which contain *Bacillus subtilis* or latex beads. Eur J Cell Biol 1995;68:167–182. 8575463

