# Supplementary material for: Differential inhibition of adenylylated and deadenylylated forms of M. tuberculosis glutamine synthetase as a drug discovery platform
Source: PLoS One. 2017 Oct 3;12(10):e0185068. doi: 10.1371/journal.pone.0185068 (PMC5626031; doi:10.1371/journal.pone.0185068)
Supplement: S1 File — (DOCX) [file pone.0185068.s001.docx]

**S1.**

**Supporting Information:**

**Preparation of Adenylylated and Deadenylylated *E. coli* GS**

**Production of Deadenylated *E.coli* GS. Plasmid construct, *E.coli* strain, transformation**: The *E.coli* YMC11 [1] strain in which the endogenous GS gene is knocked out was used as the basic expression host. To enhance the expression of fully deadenylylated *E.coli* GS, the endogenous chromosomal *E. coli* *glnE* adenylyl transferase gene was deleted using the Quick and Easy *E. coli* Gene Deletion Kit (Gene Bridges, GmbH) to create the strain YMC11E. A pBSK plasmid vector containing the *glnA* gene encoding *E.coli* GS under the transcriptional control of a constitutive T3 promoter was used to transform YMC11E by electroporation. The resultant recombinant *E. coli* strain was named pBSK-ECgln (YMC11E).

**Deadenylylated *E. coli* GS expression:** pBSKECgln (YMC11E) was used to inoculate 50 ml LM medium (1% tryptone, 0.5% yeast extract, 1% NaCl) supplemented with 50 µl Ampicillin (100 µg/ml). The inoculum was grown at 37 ºC for 16 hours with shaking at 220 rpm. Subsequently, 1ml of the culture was transferred to 50ml LM medium supplemented with 50 µl AMP_100_. This second inoculum was again grown at 37 ºC for 6 hours with shaking at 220 rpm. Subsequently, 8 ml of the culture was transferred to a modified M9 medium (6 g/l Na_2_HPO_4_, 3 g/l KH_2_PO_4_, 0.5 g/l NaCl) supplemented with 70 mM l-glutamate, trace salts (1,000x stock: 4.5 mM CaCl_2_.2H_2_O, 6.2 mM FeCl_3_.6H_2_O, 0.63 mM ZnCl_2_, 0.64 mM CuSO_4_.5H_2_O, 0.76 mM CoCl_2_.6H_2_O, 2.4 mM MnCl_2_.4H_2_O) 4% glucose, 1 mM MgSO_4_, 0.1 mM CaCl_2_ and 1 mM thiamine. The culture was grown at 37 ºC for 16 hours with shaking at 220 rpm. The cells were harvested from the cultures by centrifugation for 10 min at 16,300 *g* and the bacterial pellet used for *E. coli* GS purification.

**Production of Adenylated *E.coli* GS. Plasmid construct, *E. coli* strain, transformation:** The *E. coli* YMC11^1^ strain lacking the endogenous GS gene was used. However, to enhance the expression of fully adenylated *E. coli* GS, the endogenous *E. coli* uridylyl transferase gene (*glnD*) was deleted using the Quick and Easy *E. coli* Gene Deletion Kit (Gene Bridges, GmbH) to create the strain YMC11D. The same vector as above was introduced into YMC11D by electroporation. The resultant *E. coli* strain was named pBSK-ECgln (YMC11D).

**Adenylylated *E. coli* GS expression:** pBSK-ECgln (YMC11D) was used for the expression of adenylylated GS using a similar method to that described above for deadenylylated GS. The only difference is the addition of 5 mM L-glutamine to the M9 medium.

**Purification of Adenylylated and Deadenylylated *E. coli* GS.**

Step 1 (streptomycin sulphate precipitation): The bacterial pellet was resuspended in 10ml of RBA (10 mM imidazole pH 7, 10mM MnCl_2_) and sonicated for 20 min on a 50% on/off cycle, followed by centrifugation for 10 min at 12i100 *g* (4 ºC) to collect the soluble *E. coli* lysate. A precipitation step was carried out by adding streptomycin sulphate to a final concentration of 1% and stirring at 4 ºC for 10 min. After centrifugation for 10 min at 12,100 *g* (4 ºC) the supernatant was retained and the pH was adjusted to pH 5.15 with dilute HCl. The supernatant was stirred at 4 ºC for 15 min, followed by centrifugation at 12,100 *g* for 10min (4 ºC). The supernatant was retained, and 30% by volume of ice cold saturated (NH_4_)_2_SO_4_ solution was added. The pH was adjusted to pH 4.6, and the solution stirred at 4 ºC for 15 min, followed by centrifugation at 12,100 *g* for 10 min (4 ºC). The precipitate was resuspended in 5 ml RBA and stirred at 4 ºC for 3 hours, before the pH was adjusted to pH 5.7. Stirring continued at 4 ºC overnight, followed by centrifugation at 12,100 *g* for 10 min (4 ºC).

Step 2 (AMP affinity chromatography): A 5 ml Bed volume AMP Sepharose column (Sigma A1271 adenosine-5’-monophosphate-agarose, cross-linked 4% beaded agarose) was prepared and equilibrated with 5 column volumes of Buffer A (10 mM imidazole pH7, 10 mM MnCl_2_, 150 mM NaCl). The *E. coli* GS supernatant from Step 1 was applied and allowed to bind for 1 hour with gentle shaking at 4 ºC. The column was washed with 10 column volumes of Buffer A. The *E. coli* GS was eluted with 12 ml of Buffer B (10 mM imidazole pH7, 10 mM MnCl_2_, 450 mM NaCl, 2.5 mM ADP). Fractions were collected and assayed using the γ-glutamyl transferase assay. The fractions with *E. coli* GS activity were pooled and dialysed overnight against RBA.

**Purification of adenylylated and deadenylylated MtbGS**

Step 1 (streptomycin sulphate precipitation) **:** The bacterial pellet was resuspended in 10 ml of RBA (10 mM imidazole pH 7, 10 mM MnCl_2_) buffer and sonicated for 20 min on a 50% on/off cycle, followed by centrifugation for 10 min at 12,100*g* (4 ºC) to collect the soluble *E. coli* lysate. A precipitation step was carried out by adding streptomycin sulphate to a final concentration of 1% w/v and stirring at 4 ºC for 10 min. After centrifugation for 10 min at 12,100*g* (4 ºC), the supernatant was retained and the pH was adjusted to pH 4.4 with dilute HCl. The supernatant was slowly stirred at 4 ºC overnight, followed by centrifugation at 12,100*g* (4 ºC) for 10 min and aspiration of the supernatant.

Step 2 (anion-exchange chromatography): A column containing 5 ml bed volume DEAE Sepharose CL-6B (Pharmacia Biotech) was prepared and equilibrated with 5 column volumes of Buffer H (50 mM HEPES pH 7.6, 10 mM MnCl_2_) containing 100 mM NaCl. The final supernatant from Step 1 was applied and the column washed with 10 column volumes of Buffer H containing 100 mM NaCl. Bound *Mtb*GS was eluted by applying a step gradient of Buffer H containing 0.4 M, 0.5 M, 0.6 M and 0.7 M NaCl. Fractions were collected and assayed for the presence of *Mtb*GS using the γ-glutamyl transferase assay. The fractions with GS activity were pooled and dialysed overnight against Buffer H containing 100 mM NaCl.

Step 3 (AMP affinity chromatography): A column containing 5 ml bed volume AMP Sepharose (Sigma A1271 adenosine-5’-monophosphate-agarose, cross-linked 4% beaded agarose) was prepared and equilibrated with 5 column volumes of Buffer A (10 mM imidazole pH 7.0, 10 mM MnCl_2_, 150 mM NaCl). The pooled *Mtb*GS fractions from Step 2 were applied and allowed to bind for 1 ½ hours with gentle shaking at 4 ºC. The column was washed with 10 column volumes Buffer A. *Mtb*GS was eluted with 10 ml of Buffer B (10 mM imidazole pH 7.0, 10 mM MnCl_2_, 450 mM NaCl, 2.5 mM ADP). Fractions were collected and the fractions with γ-glutamyl transferase activity were pooled and dialysed overnight against Buffer H containing 100 mM NaCl.

Sodium dodecyl-sulphate polyacrylamide gel electrophoresis (SDS-PAGE) was used to analyse the molecular mass and purity of the isolated enzymes [42]. Protein concentrations were determined by using the Quant-IT^TM^ Protein Assay Kit (Invitrogen, USA) used in conjunction with the QUBIT^TM^ fluorometer.

**Reference**

1. Backman K Chen Y-M, Magasanik B. 1981. Physical and genetic characterization of the *glnA*-*glnG* region of the *Escherichia* *coli* chromosome. Proc Natl Acad Sci USA. 78:3743-3747.
